# Experimental Study on Mechanical Property of Cemented Backfill in Coal Mine

**DOI:** 10.3390/ma18184423

**Published:** 2025-09-22

**Authors:** Haigang Yang, Rui Wang, Qiang Zhang, Wencheng Ma, Yukai Wang

**Affiliations:** 1 Key Laboratory of Xinjiang Coal Resources Green Mining, Ministry of Education, Xinjiang Institute of Engineering, Urumqi 830023, China; 2 School of Mining Engineering and Geology, Xinjiang Institute of Engineering, Urumqi 830023, China; 3 Chief Engineer’s Office, Xinjiang Yihua Mining Co., Ltd., Changji 831100, China; 4 Wucaiwan No.1 Open-Pit Coal Mine, Xinjiang Yihua Mining Co., Ltd., Changji 831100, China; 5 School of Civil Engineering, North China University of Technology, Beijing 100144, China

**Keywords:** cemented backfill in coal mine, shear strength, slurry concentration, segregation degree, homogeneity

## Abstract

In response to the insufficiency of shear strength and severe segregation of cemented backfilling material in coal mines, a shear strength test, static segregation index test, and homogeneity degree test were carried out, taking slurry concentration (SC) as the main control factor. The effect law of SC on shear strength, the static segregation index, and the homogeneity degree was discussed. The relationship between the static segregation index and homogeneity degree and shear strength was analyzed, and the action mechanism of SC on shear strength was revealed. The research results show that for cemented backfill in coal mines, with a suspending agent content of 0 and a curing age of 28 d, when SC increases from 77% to 80%, shear strength increases by 31.43%, the static segregation index of the backfilling slurry decreases by 40.29%, and the homogeneity degree of the backfill increases by 69.23%. The increase in SC can enhance shear strength, reduce the segregation degree of backfilling slurry, and improve the homogeneity of backfill. The reason for the increase in shear strength lies in the fact that SC reduces the segregation degree of the backfilling slurry. The research in this paper has certain guiding significance for the timely support of the surrounding rock in the working face and the effective control of surface settlement.

## 1. Introduction

The implementation of backfilling mining in coal mines, firstly, can restrict the rupture and spillage of the immediate and main roof above the already-mined coal seams, effectively suppress the development height of the fracture zone, and prevent the occurrence of surface cracking and collapse [1,2]. Secondly, the goaf left behind after the propulsion of the coal mining face is backfilled, which can effectively inhibit the formation of water diversion channels [3,4]. Thirdly, it avoids the spontaneous combustion of the coal left in the goaf and the desorption and emission of a large amount of toxic and harmful gases, reducing the direct emission of greenhouse gases, like gas [5,6]. Finally, backfilling mining in coal mines eliminates gangue hills and fly ash piles and reduces harm to surface water bodies, soil, air, and living spaces [7,8,9,10].

Due to the fact that cemented backfilling slurry in coal mines is prone to segregation, the backfill strength of cemented backfill in coal mines (CBCM) formed by coagulation drops significantly, which seriously affects the effect of backfilling mining [11,12]. When the easily segregated backfilling slurry is placed in the goaf, it is difficult for backfill to reach the expected strength within the prescribed time, which is not conducive to maintaining the stability of the surrounding rock of the stope. If the mining time is sacrificed to increase backfill strength, it will seriously affect the normal propulsion of the mining face. In addition, the long-term strength of CBCM is also limited, making it difficult to keep the surface settlement within a reasonable range and wasting the capital investment in goaf management. In order to normally exert the load-bearing function of CBCM, it is necessary to improve the segregation performance of cemented backfilling slurry in coal mines.

Similarly to compressive strength and flexural strength, shear strength is also one of the important mechanical properties of backfill. The failure forms of geotechnical materials are essentially shear failure and tensile failure. When the backfill coagulating into the goaf is subjected to shear loads, shear failure will occur. The shear performance of backfill has increasingly drawn the attention of experts and scholars [13,14,15].

In view of the phenomenon that cement-based materials are prone to segregation, many experts and scholars have conducted extensive research and achieved many valuable research results. Common influencing factors of segregation behavior include types and contents of binder [16,17], aggregate gradation [18,19], fly ash content [20,21], types and contents of aggregate [22,23], types and contents of admixture [24,25] and slurry concentration (SC) [26,27,28,29,30,31], etc. The change in SC has little impact on the cost of backfilling mining in coal mines and can significantly reduce segregation behavior of backfilling slurry. Peng [26] discussed the variation trend of anti-segregation performance of tailings backfill slurry with SC. With the increase in SC, the anti-segregation performance of tailings backfill slurry enhances. Chen et al. [27] analyzed the coupling effects of inlet velocity, particle mass concentration, and particle size of cemented paste backfill material. Wang and Gan [28] developed a pipeline transport model to analyze the effects of SC, particle size and velocity on particle settlement rate. Wang et al. [29] evaluated the anti-segregation performance of coarse aggregate paste by an orthogonal test. Li [30] studied the effects of the amount of fly ash, the ratio of fine gangue, and mass concentration on the static anti-segregation performance of coal gangue cemented backfill materials by the response surface method. Li et al. [31] constructed the anti-segregation performance model based on yield stress, particle gradation, and SC and verified the rationality of the theoretical model.

As the effect law and action mechanism of SC on the shear strength of CBCM is still unclear, the relevant research of this paper has been carried out. This paper discussed the effect of SC on the shear strength, static segregation index, and homogeneity degree of CBCM and analyzed the relationship between the static segregation index, homogeneity degree, and shear strength. Placing CBCM with sufficient shear strength in the goaf is conducive to maintaining the stability of the surrounding rock of the mining area in the short term. In the long run, the backfill can effectively limit the surface settlement.

## 2. Materials and Methods

### 2.1. Raw Materials

The coal gangue was selected from the gangue hill of a coal mine of Kailuan Group (Tangshan, China), Its density is 2.3–2.5 g/cm^3^. The mineral components are mainly quartz, kaolinite, and white mica. The chemical composition of coal gangue was tested by the PANalytical Axios X-ray fluorescence spectrometer (Malvern Panalytical Company, Shanghai, China), and the test results are shown in Table 1.

**Figure 1:**
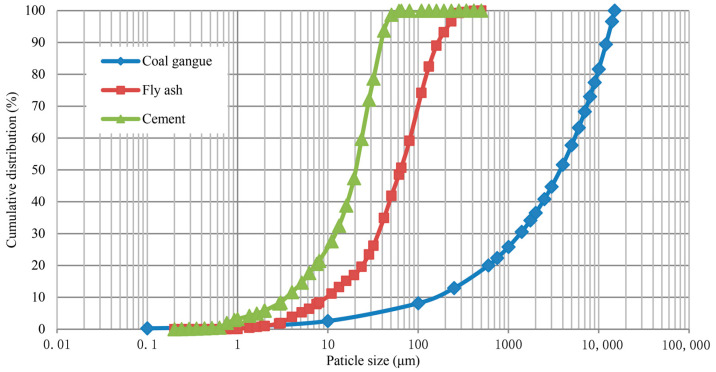
Particle size distribution of raw materials.

The aggregate gradation has a significant impact on the shear strength. To avoid interference with test results from the aggregate gradation, the gradation reduction coefficient of coal gangue was determined to be 0.5. The particle size distribution of coal gangue is shown in Figure 1.

**Figure 2:**
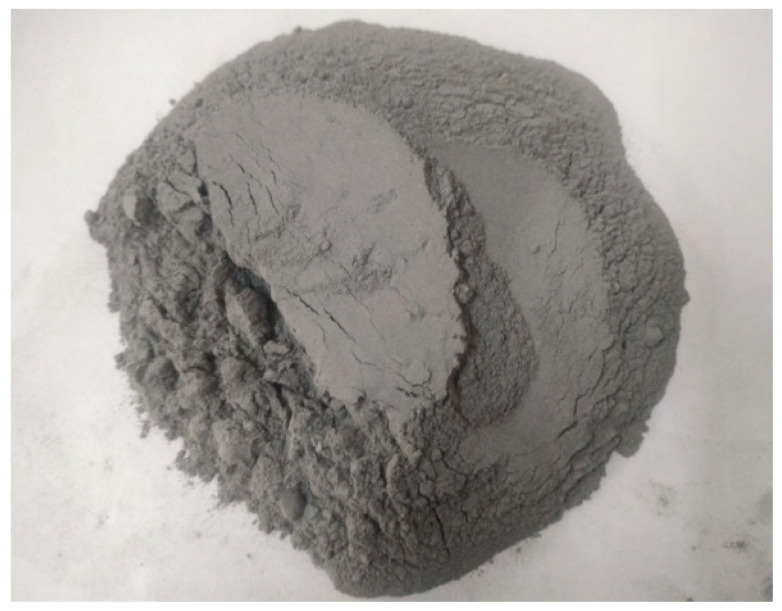
The morphology of fly ash.

The fly ash was selected from a coal-fired power plant near a coal mine of Kailuan Group. Its density is 1.8 to 2.8 g/cm^3^, and the specific surface area is 2200 to 4000 cm^2^/g. The chemical composition test of fly ash was conducted on the PANalytical Axios X-ray fluorescence spectrometer using the pellet method. The test results are shown in Table 1. The particle size of fly ash was tested by the LS-C (II A) laser particle size analyzer (Zhuhai OMEC Instrument Co., Ltd., Zhuhai, Guangdong, China), and the test results are shown in Figure 1. The characteristic parameters of particle size are as follows: *d*_30_ is 36.38 μm, *d*_50_ is 63.81 μm, and *d*_90_ is 165.82 μm. The specific morphology of fly ash is shown in Figure 2.

The cement was selected from ordinary Portland cement sold in the market. Its density is 3.03 g/cm^3^, the specific surface area is 340 m^2^/kg, and the initial setting time is 165 min. The chemical composition of cement is shown in Table 1. The particle size test of cement was carried out on the LS-C (II A) laser particle size analyzer. The test results are shown in Figure 1. The characteristic parameters of particle size are as follows: *d*_30_ is 12.14 μm, *d*_50_ is 20.30 μm, and *d*_90_ is 39.08 μm.

The mixing water consisted of clean tap water from the laboratory, whose quality meets the relevant requirements of the JGJ63-2006 concrete water standard [32].

Taking into account the effect of the pre-test results, dissolution rate, market price, and other indicators comprehensively, hydroxypropyl methylcellulose produced by a chemical technology company in Shanghai, China, was selected as the suspending agent for slurry preparation. The viscosity of hydroxypropyl methylcellulose is 200,000, apparent density ranges from 0.25 to 0.70 g/cm^3^, and its form is a white or white-like fiber or granular powder.

### 2.2. Test Method

#### 2.2.1. Shear Strength Test

**Figure 3:**
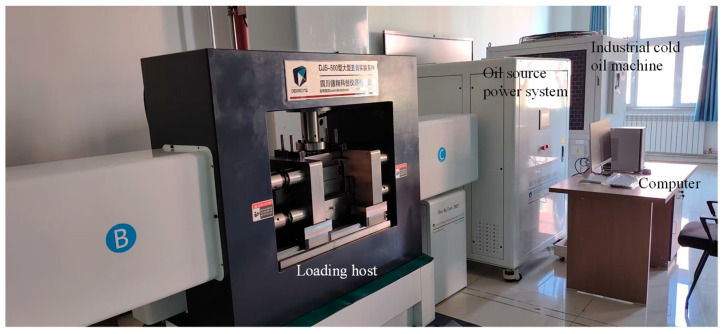
DJS-500 large-scale direct shear test system.

This shear strength test was conducted on the DJS-500 large-scale direct shear test system (Sichuan dexkcyq Instrument Co., Ltd., Chengdu, Sichuan, China). This system is mainly composed of an industrial cold oil machine, an oil source power system, a loading host, a computer, and compression accessories, as shown in Figure 3. The maximum load for vertical and horizontal loading is 500 kN, and the force loading rate in both directions is 0.01 to 20 kN/s. The maximum displacement for vertical and horizontal loading is 100 mm, and the displacement loading rate of the oil cylinder in both directions is 0.1 to 200 mm/min. The measurement range of vertical and horizontal displacement is 0 to 12.5 mm.

Fly ash has a strong hygroscopicity. The seemingly dry fly ash actually stores a large amount of moisture. Direct use in the preparation of backfilling slurry will lead to a significant decrease in SC. Therefore, fly ash needs to be dried before the test. The specific operation is to place the fly ash after screening and impurity removal in a drying oven at 65 °C for 48 h. The preparation of backfilling slurry strictly followed GB/T50080-2016 [33]. The sample size is 100 mm × 100 mm × 100 mm (length × width × height), and normal stress is 0 MPa. The test steps for the shear strength test referred to GB/T23561-2024 [34].

The shear strength (*τ*) of CBCM was tested with SC (*η*) as the main control factor and suspending agent content (*ω*) and curing age (*A*) as the auxiliary factors. The test scheme is shown in Table 2. Suspending agent content is the proportion of the total mass of the backfilling slurry in Table 2. The curing humidity is above 95%.

#### 2.2.2. Static Segregation Index Test

**Figure 4:**
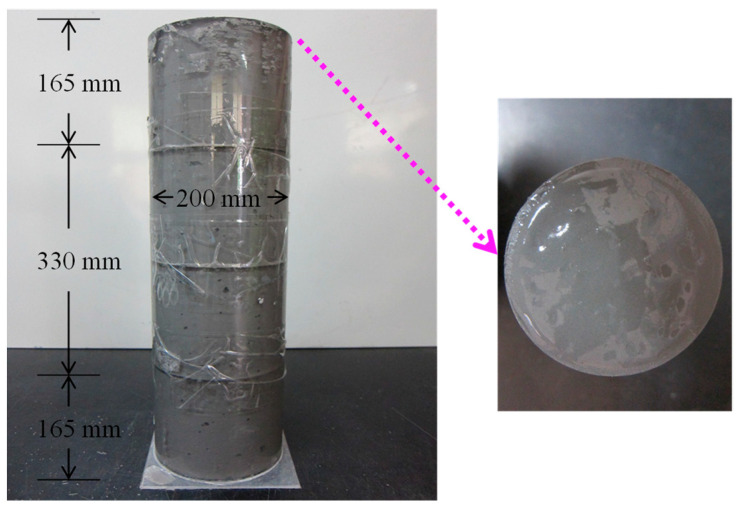
Test mold of static segregation index.

There are many testing methods for the segregation performance of cement-based materials, including both qualitative and quantitative ones [30]. We mainly use the columnar method to test the segregation performance of cemented backfilling slurry in coal mines. The testing process strictly followed ASTM C1610-14 [35]. The test cylinder is divided into three layers by height. The top and bottom layers are 165 mm high, the middle layer is 330 mm high, and the nominal diameter is 200 mm, as shown in Figure 4. The relevant operation steps are as follows: (1) pour the prepared backfilling slurry into the test cylinder; (2) let the slurry in the cylinder stand for (15 ± 1) minutes; (3) place the top slurry and bottom slurry, respectively, on 4.75 mm square-hole sieves and rinse them; (4) place the coarse gangue on the square-hole screen in a drying oven at 65 °C for 48 h; and (5) weigh the dried coarse gangue.

The static segregation index was calculated by Equation (1). From Equation (1), it can be seen that the smaller the static segregation index, the lower the segregation degree of backfilling slurry, and the more dispersed the various solid particles in the slurry.

(1) $$SI=\frac{m_{b}-m_{t}}{(m_{b}+m_{t})/2}$$

where *SI* is the static segregation index (%); *m*_b_ is the mass of coarse gangue in the bottom slurry (kg); and *m*_t_ is the mass of coarse gangue in the top slurry (kg).

The backfilling slurry with a suspending agent content of 0 was selected for the static segregation index test, and SC was 77%, 78%, 79%, and 80%, respectively. The test scheme is shown in Table 3.

#### 2.2.3. Homogeneity Degree Test

**Figure 5:**
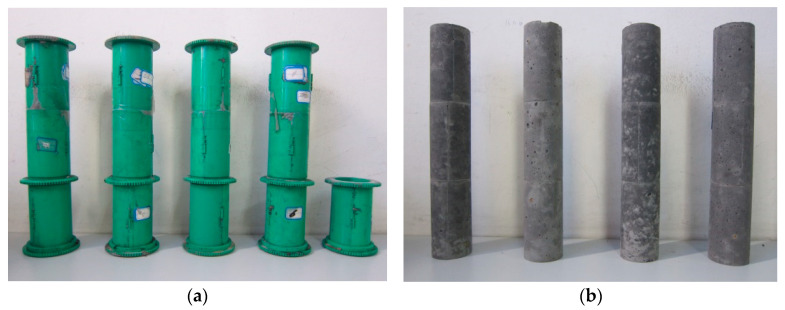
Test mold of homogeneity degree: (**a**) molds; (**b**) samples.

This homogeneity degree test was carried out on the YDW-50 microcomputer-controlled electronic pressure testing machine (Jinan Wenteng Testing Instrument Co., Ltd., Jinan, Shandong, China). Three test molds with a size of *Φ*50 × 100 mm were spliced to obtain one mold with a size of *Φ*50 × 300 mm, as shown in Figure 5a. The molds were poured with the prepared backfilling slurry, and the samples after curing are shown in Figure 5b.

The sample with a size of *Φ*50 × 300 mm was divided into three equal parts with a ruler and then cut on the cutting machine according to the division lines. The uniaxial compressive strength tests of the top and bottom backfill were carried out in strict accordance with GB/T50081-2019 [36].

The ratio of the uniaxial compressive strength of the bottom backfill to that of the top backfill was defined as the homogeneity degree, as shown in Equation (2). From Equation (2), it can be seen that the greater the homogeneity degree, the higher the homogeneity of the backfill, and the more dispersed the various solid particles in backfill.

(2) $$HI=\frac{\sigma_{b}}{\sigma_{t}}$$

where *HI* is the homogeneity degree (%); *σ*_b_ is the uniaxial compressive strength of the bottom backfill (MPa); and *σ*_t_ is the uniaxial compressive strength of the top backfill (MPa).

The backfill with a suspending agent content of 0 and a curing age of 28 d was selected for the homogeneity degree test, and SC was 77%, 78%, 79% and 80%, respectively. The test scheme is shown in Table 4.

## 3. Results

### 3.1. Effect of SC on Shear Strength

**Figure 6:**
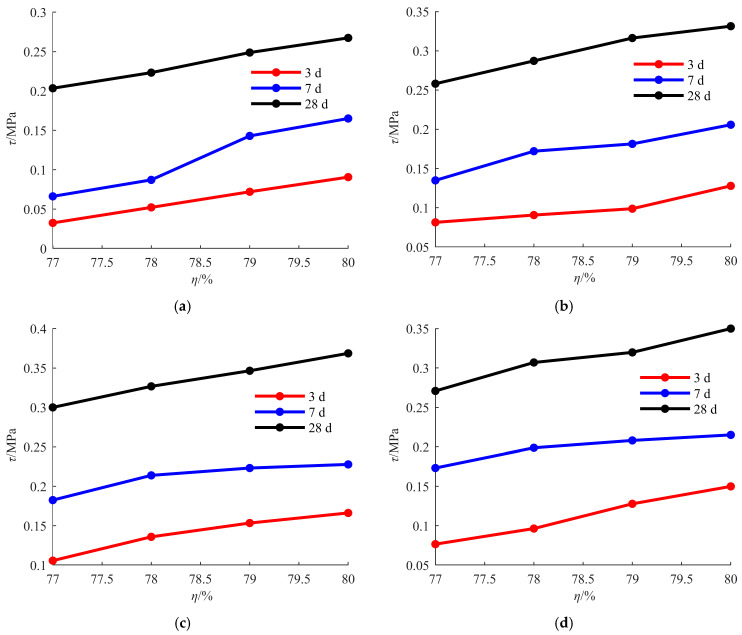
Change curve of shear strength with SC: (**a**) *ω* = 0; (**b**) *ω* = 0.02%; (**c**) *ω* = 0.04%; and (**d**) *ω* = 0.06%.

The change curve of shear strength with SC is shown in Figure 6.

Shear strength under the same suspending agent contents and the same curing ages shows an increasing trend with the increase in SC, as shown in Figure 6. Taking the backfill with a suspending agent content of 0 and a curing age of 28 d as an example, the change law of shear strength is analyzed. When SC increases from 77% to 78%, shear strength rises from 0.2035 MPa to 0.2233 MPa, with an increase of 9.71%. The growth rate of shear strength is close to 10%, which means that SC has a promoting effect on shear strength. When SC increases from 78% to 79%, shear strength rises from 0.2233 MPa to 0.2488 MPa, with an increase of 11.46%. The growth rate of shear strength becomes larger, indicating that SC has a greater promoting effect on shear strength within this range. When SC increases from 79% to 80%, shear strength rises from 0.2488 MPa to 0.2674 MPa, with an increase of 7.48%. Although the growth rate of shear strength slows down within this range, SC still has a promoting effect on shear strength. When SC changes from 77% to 80%, the overall growth rate of shear strength is 31.43%. Under the different suspending agent contents, with the increase in curing age, the change trend of shear strength with SC remains approximately unchanged, indicating that the effect trend of SC on shear strength is not affected by suspending agent content and curing age.

### 3.2. Effect of SC on Static Segregation Index

**Figure 7:**
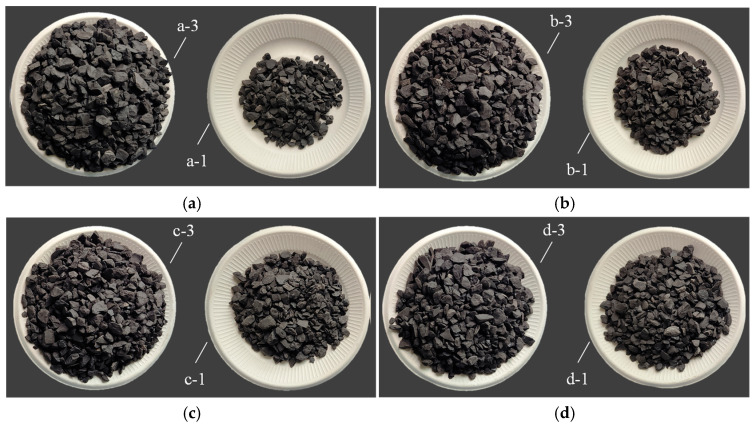
Test results of static segregation index: (**a**) *η* = 77%; (**b**) *η* = 78%; (**c**) *η* = 79%; and (**d**) *η* = 80%.

The test results of the static segregation index are shown in Figure 7. In Figure 7, “1” represents the top of the backfilling slurry in Figure 4, and “3” in Figure 7 represents the bottom of backfilling slurry in Figure 4.

**Figure 8:**
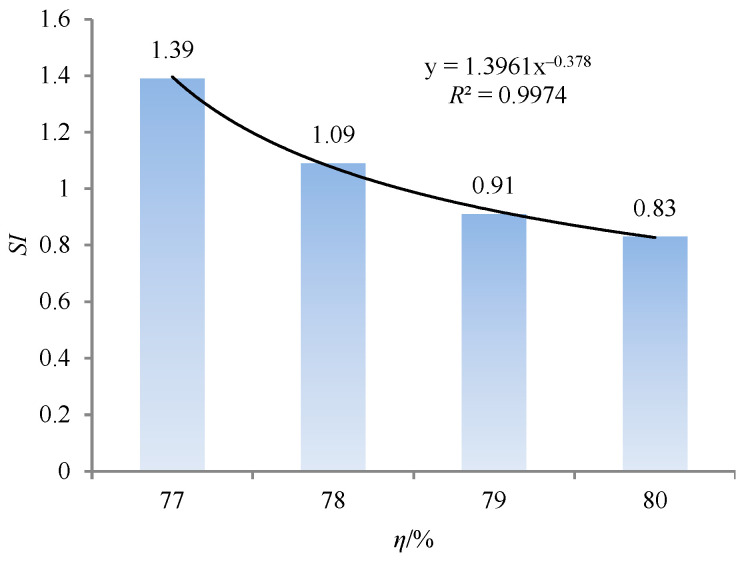
Change curve of static segregation index with SC.

The mass of coarse gangue in the top slurry is different from that in the bottom slurry, as shown in Figure 7. When SC is 77%, the mass of coarse gangue in the top slurry is 0.58 kg, and that in the bottom slurry is 3.25 kg. When SC is 80%, the mass of coarse gangue in the top slurry is 1.15 kg, and that in the bottom slurry is 2.77 kg. With the increase in SC, the mass of the coarse gangue in the top slurry gradually approaches that in the bottom slurry. The change curve of the static segregation index with SC is shown in Figure 8.

As shown in Figure 8, the static segregation index is 1.39 when SC is 77%. The static segregation index is 0.83 when SC is 80%, with a decrease of 40.29%. There is a power function relationship between SC and the static segregation index (see Figure 8). The static segregation index of the backfilling slurry decreases, indicating that the segregation degree of the backfilling slurry decreases. The increase in SC is achieved by reducing moisture and increasing the number of solid particles, thereby enhancing the consistency of the backfilling slurry. The increase in consistency, on the one hand, will lead to a reduction in the settling space of solid particles, and on the other hand, it will also increase the friction between solid particles, making it difficult for similar substances to aggregate, thereby maintaining the dispersibility of solid particles. In addition, according to the research of Wang et al. [29], an increase in SC will lead to an increase in yield stress, which is directly proportional to viscous resistance. Therefore, an increase in SC will cause solid particles to encounter greater viscous resistance during the descent. This resistance can effectively slow down the settling rate of various solid particles, prevent the aggregation of similar substances, and maintain the dispersion of various solid particles at the initial stage of preparation. Therefore, under the combined effect of consistency and yield stress, SC effectively reduces the segregation degree of the backfilling slurry.

Not only is the mass of coarse gangue changing, but its size is also changing. With the increase in SC, the particle size difference between coarse gangue in the top slurry and that in the bottom slurry decreases, as shown in Figure 7. Large coarse gangue particles gradually appear in the top slurry, further indicating that the difference between the top slurry and the bottom slurry decreases. The backfilling slurry transforms from segregation to non-segregation; that is, the segregation degree of the backfilling slurry decreases.

### 3.3. Effect of SC on Homogeneity Degree

**Figure 9:**
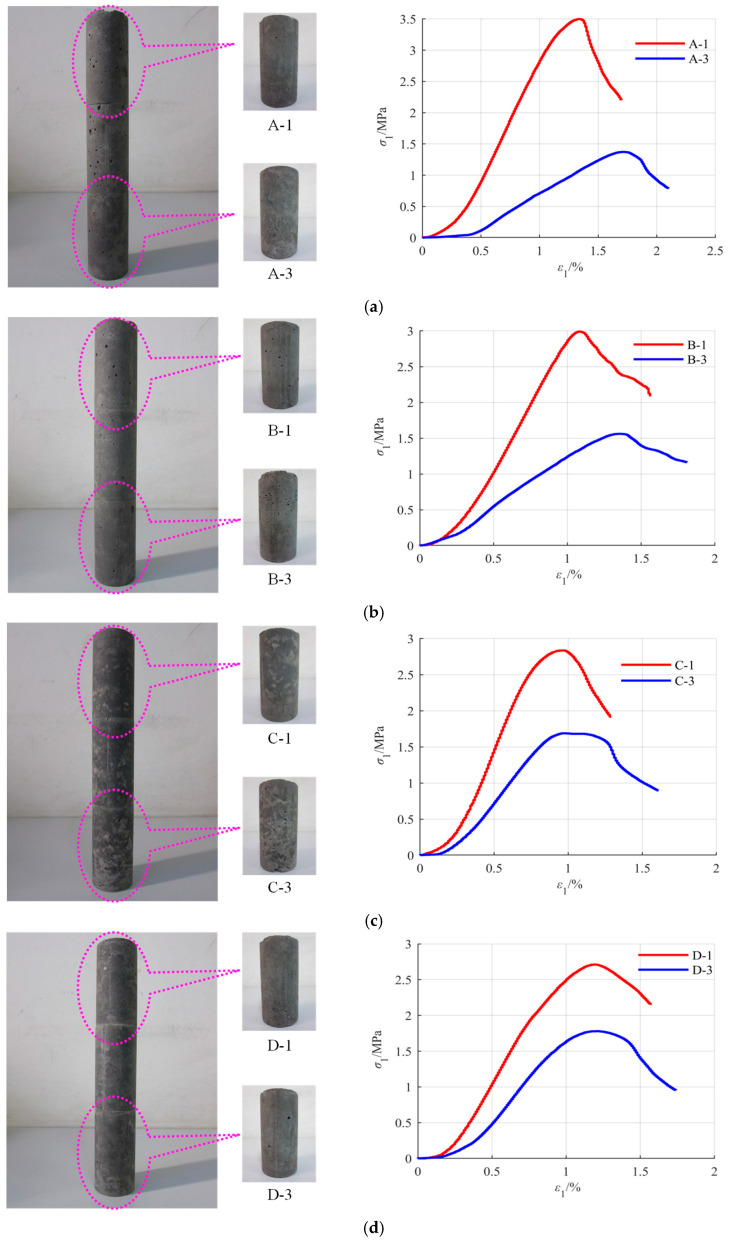
Test results of homogeneity degree: (**a**) *η* = 77%; (**b**) *η* = 78%; (**c**) *η* = 79%; and (**d**) *η* = 80%.

The test results of the homogeneity degree are shown in Figure 9. In Figure 9, “1” represents the top of backfill, and “3” represents the bottom of backfill.

**Figure 10:**
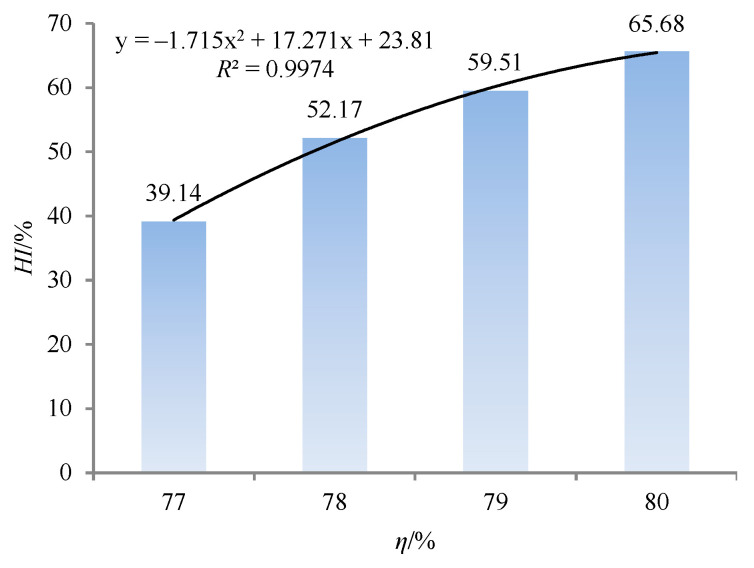
Change curve of homogeneity degree with SC.

The uniaxial compressive strength of the top backfill is different from that of the bottom backfill, as shown in Figure 9. When SC is 77%, the uniaxial compressive strength of the top backfill is 3.50 MPa, and that of the bottom backfill is 1.37 MPa. When SC is 80%, the uniaxial compressive strength of the top backfill is 2.71 MPa, and that of the bottom backfill is 1.78 MPa. With the increase in SC, the uniaxial compressive strength of the top backfill gradually approaches that of the bottom backfill. The change curve of the homogeneity degree with SC is shown in Figure 10.

As shown in Figure 10, the homogeneity degree is 39.14% when SC is 77%. The homogeneity degree is 65.68% when SC is 80%, with an increase of 69.23%. There is a quadratic function relationship between SC and the homogeneity degree (see Figure 10). The homogeneity degree of backfill increases, indicating that the homogeneity of backfill improves. SC changes the segregation degree of the backfilling slurry through consistency and yield stress. If the segregation degree of the backfilling slurry is low, the homogeneity of the backfill formed later will be high. Therefore, it can also be considered that SC improves the homogeneity of the backfill through consistency and yield stress.

Not only is the uniaxial compressive strength changing, but the pre-peak elastic modulus and the morphology of the stress–strain curve are also changing. With the increase in SC, the difference in the stress–strain curves between the top backfill and the bottom backfill decreases, as shown in Figure 9. The pre-peak elastic modulus and curve morphology both show great similarity, further indicating that the difference between the top backfill and the bottom backfill decreases; that is, the homogeneity of the backfill improves.

## 4. Discussion

### 4.1. Relationship Between Static Segregation Index and Shear Strength

**Figure 11:**
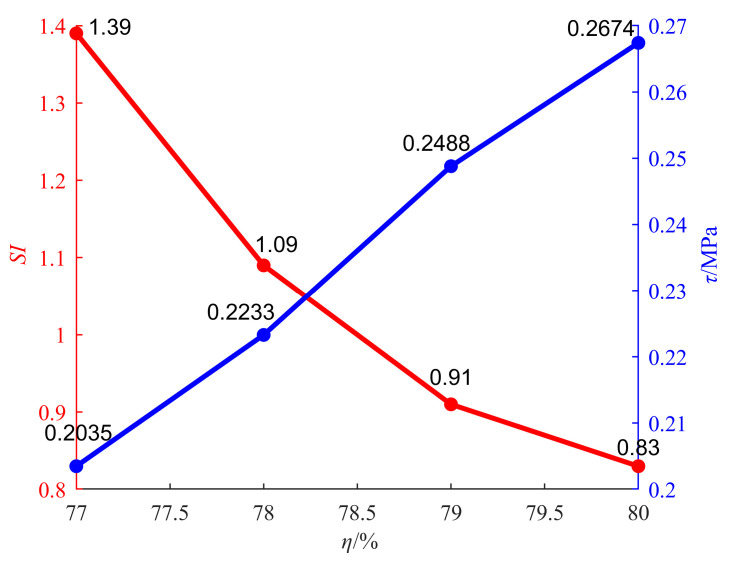
Relationship between static segregation index and shear strength.

The change curve of the static segregation index with SC is shown as the red curve in Figure 11, and the change curve of 28 d shear strength with SC is shown as the blue curve in Figure 11.

The red curve in Figure 11 shows that the static segregation index of backfilling slurry decreases with the increase in SC. The blue curve in Figure 11 shows that shear strength increases with the increase in SC. The increase in shear strength is mainly due to the fact that SC reduces the segregation degree of the backfilling slurry. As the segregation degree of backfilling slurry decreases, various solid particles inside the backfill are evenly distributed. It is difficult to form a weak structural surface inside the backfill. When the backfill is subjected to external loads, it is not prone to cracking. Higher integrity ensures the continuous transmission of stress within the backfill, thereby demonstrating a higher resistance to external loads. Macroscopically, this is reflected in the increase in shear strength.

### 4.2. Relationship Between Homogeneity Degree and Shear Strength

**Figure 12:**
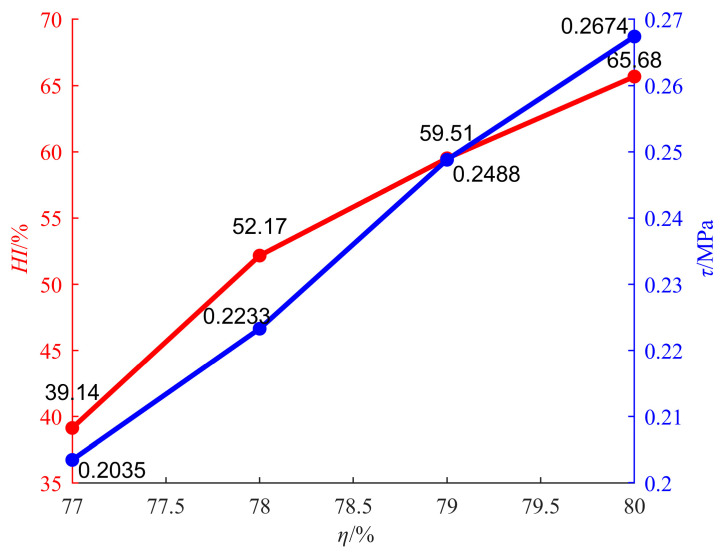
Relationship between homogeneity degree and shear strength.

The change curve of the homogeneity degree with SC is shown as the red curve in Figure 12, and the change curve of 28 d shear strength with SC is shown as the blue curve in Figure 12.

The red curve in Figure 12 shows that the homogeneity degree of the backfill increases with the increase in SC. The blue curve in Figure 12 shows that shear strength increases with the increase in SC. The increase in shear strength is mainly due to the fact that SC enhances the homogeneity of backfill. For cemented backfilling materials in coal mines, the homogeneity and segregation degree are both used to describe the dispersion characteristics of particles such as coal gangue, fly ash, and unhydrated cement in the backfilling materials. However, homogeneity is mainly used to characterize the dispersion characteristics in the set state, while the segregation degree is mainly used to characterize the dispersion characteristics in the slurry state. The lower the segregation degree of backfilling slurry, the more evenly the particles such as coal gangue, fly ash, and unhydrated cement in the backfilling material are dispersed, and the higher the homogeneity of the backfill formed by coagulation. Therefore, the action mechanism of homogeneity on shear strength is the same as that of the segregation degree on shear strength. Here, the reasons for the change in shear strength with homogeneity will not be elaborated. While the test results of the homogeneity degree are mutually corroborated with those of the static segregation index, the action mechanism of SC on shear strength is further verified.

## 5. Conclusions

This paper took SC as the main control factor and analyzed the influence law of SC on shear strength of CBCM. The reasons for the variation in shear strength with SC were explained by means of the static segregation index test and homogeneity degree test. The main conclusions obtained are as follows.

1. For CBCM with the same suspending agent content and the same curing age, shear strength increases with the increase in SC. SC has a significant promoting effect on shear strength. The effect trend of SC on shear strength is not affected by suspending agent content and curing age.
2. With the increase in SC, the mass of coarse gangue in the top slurry increases while that in the bottom slurry decreases. The particle size of the coarse gangue in the top slurry gradually approaches that in the bottom slurry. The uniaxial compressive strength of the top backfill drops while that of the bottom backfill rises. The pre-peak elastic modulus and the morphology of the stress–strain curve of the top backfill gradually approach that of the bottom backfill. SC can significantly reduce the segregation degree of the backfilling slurry and improve the homogeneity of the backfill.
3. The reduction in the segregation degree of the backfilling slurry or the increase in the homogeneity of the backfill can improve the shear strength of the backfill. The lower the segregation degree of the backfilling slurry, the less likely the backfill formed after coagulation is to crack, which enables the backfill to exhibit a greater load-bearing capacity.

Adequate shear strength can stabilize the surrounding rock in the short term and limit surface settlement in the long term. Although SC and suspending agent content have changed, cemented backfilling material in coal mines involved in this paper still has good rheological properties [37,38], setting time, and working performance [24], which can meet the requirements of pipeline transportation and timely bearing. Therefore, the above properties are not studied in detail in this paper. Fly ash and cement are important building materials and play an indispensable role in production and daily life. Some types of fly ash and cement have certain radioactivity [39,40]. This issue has not been discussed in this article so far. In subsequent research, we will pay more attention to the radioactivity of fly ash, cement, and even coal gangue.

## Figures and Tables

**Table 1:** Chemical composition of raw materials.

Raw Materials	Chemical Composition/%							
	SiO_2_	CaO	Al_2_O_3_	Fe_2_O_3_	SO_3_	TiO_2_	K_2_O	Other
Coal gangue	34.62	28.44	21.38	6.49	4.85	1.12	0.63	2.47
Fly ash	54.42	0.98	31.52	5.36	0.84	1.56	1.39	3.93
Cement	25.12	42.35	12.11	3.98	6.45	3.95	0.22	5.82

**Table 2:** Test scheme of shear strength.

*η* (%)	*ω* (%)	Material Mix Ratio/%	*t* (°C)	*A* (d)
		Coal Gangue:Fly Ash:Cement:Water		
77	0, 0.02, 0.04 and 0.06	47:20:10:23	20 ± 2	3, 7 and 28
78		48:20:10:22		
79		49:20:10:21		
80		50:20:10:20		

**Table 3:** Test scheme of static segregation index.

*ω* (%)	*η* (%)	Material Mix Ratio/%	*t* (°C)	*A* (min)
		Coal Gangue:Fly Ash:Cement:Water		
0	77	47:20:10:23	20 ± 2	15
	78	48:20:10:22		
	79	49:20:10:21		
	80	50:20:10:20		

**Table 4:** Test scheme of homogeneity degree.

*ω* (%)	*η* (%)	Material Mix Ratio/%	*t* (°C)	*A* (d)
		Coal Gangue:Fly Ash:Cement:Water		
0	77	47:20:10:23	20 ± 2	28
	78	48:20:10:22		
	79	49:20:10:21		
	80	50:20:10:20		

## Data Availability

The raw data supporting the conclusions of this article will be made available by the authors on request.

## Abbreviations

The following abbreviations are used in this manuscript:

CBCM	Cemented backfill in coal mine
SC	Slurry concentration
